# Hydrogen sulfide stimulates CFTR in *Xenopus* oocytes by activation of the cAMP/PKA signalling axis

**DOI:** 10.1038/s41598-017-03742-5

**Published:** 2017-06-14

**Authors:** Alexander Perniss, Kathrin Preiss, Marcel Nier, Mike Althaus

**Affiliations:** 1 Institute for Animal Physiology, Justus-Liebig-University, Giessen, Germany; 2 Present Address: Institute for Anatomy and Cell Biology, Justus-Liebig-University, Giessen, Germany; 3 School of Biology, Newcastle University, Newcastle upon Tyne, United Kingdom

## Abstract

Hydrogen sulfide (H_2_S) has been recognized as a signalling molecule which affects the activity of ion channels and transporters in epithelial cells. The cystic fibrosis transmembrane conductance regulator (CFTR) is an epithelial anion channel and a key regulator of electrolyte and fluid homeostasis. In this study, we investigated the regulation of CFTR by H_2_S. Human CFTR was heterologously expressed in *Xenopus* oocytes and its activity was electrophysiologically measured by microelectrode recordings. The H_2_S-forming sulphur salt Na_2_S as well as the slow-releasing H_2_S-liberating compound GYY4137 increased transmembrane currents of CFTR-expressing oocytes. Na_2_S had no effect on native, non-injected oocytes. The effect of Na_2_S was blocked by the CFTR inhibitor CFTR_inh172, the adenylyl cyclase inhibitor MDL 12330A, and the protein kinase A antagonist cAMPS-Rp. Na_2_S potentiated CFTR stimulation by forskolin, but not that by IBMX. Na_2_S enhanced CFTR stimulation by membrane-permeable 8Br-cAMP under inhibition of adenylyl cyclase-mediated cAMP production by MDL 12330A. These data indicate that H_2_S activates CFTR in *Xenopus* oocytes by inhibiting phosphodiesterase activity and subsequent stimulation of CFTR by cAMP-dependent protein kinase A. In epithelia, an increased CFTR activity may correspond to a pro-secretory response to H_2_S which may be endogenously produced by the epithelium or H_2_S-generating microflora.

## Introduction

The cystic fibrosis transmembrane conductance regulator (CFTR) is a chloride and bicarbonate conducting anion channel which is found in many vertebrate epithelia and essential for the epithelial regulation of electrolyte and fluid homeostasis. Gene mutations in CFTR cause cystic fibrosis, the most common autosomal-recessive disorder in Caucasians, with a disease incidence of around 1 in 1000–3000 in northern Europeans^1^. The CFTR protein contains two membrane-spanning regions (each consisting of six transmembrane domains) functioning as the channel pore, which are connected to two intracellular nucleotide binding domains (NBD1 and NBD2) as well as a regulatory (R) domain^2^. NBD1 and NBD2 regulate the opening and closing of the channel by binding and hydrolysing ATP^2,3^, whereas the R-domain initiates the transitions in channel conformation by protein kinase A (PKA)-dependent phosphorylation^2^. Although the NBDs and R-domain contain various phosphorylation sites which control biogenesis, trafficking, interaction with other proteins and channel open probability^2^, PKA is the primary regulator of CFTR activity. At the cellular level, CFTR is hence mainly regulated by cAMP-coupled signalling events.

CFTR is located in the apical membrane of epithelial cells, where it primarily represents a conductance for chloride ions and facilitates transepithelial movement of chloride^4^. CFTR allows chloride permeation in and out of the cells and the direction of chloride flux is determined by the gradient for chloride ions between the cytoplasm and luminal extracellular fluid, as well as the apical membrane potential of the epithelial cells^5^. Whereas CFTR mediates chloride secretion in the intestine, pancreas, secretory coils of sweat glands or serous cells of airway submucosal glands^4,6^, it absorbs chloride in the ducts of sweat glands^4^ or, as recently suggested, in the airway surface epithelium^7^. Its physiological importance is also reflected in the consequence of CFTR malfunction and cystic fibrosis phenotype which includes pancreas insufficiency, airway mucus obstruction, meconium ileus and high sweat chloride concentrations^1^. Under physiological conditions, multiple cellular signalling cascades regulate the activity or membrane abundance of CFTR^8^, thus allowing for a precise regulation of chloride flux and, eventually, electrolyte and fluid homeostasis.

Hydrogen sulfide (H_2_S) is a well-known environmental chemical threat with a characteristic odour of rotten eggs; however, research over the past decade has revealed that H_2_S is also an important cellular signalling molecule^9^. H_2_S is involved in a variety of physiological and patho-physiological processes (for review see ref.^9^), and H_2_S-liberating compounds are currently explored for a therapeutic potential^10^. Physiological concentrations of H_2_S are likely in the nano- to low micro-molar range and depend on its production, intracellular storage and mitochondrial degradation^11^. H_2_S is enzymatically generated within the metabolism of L-cysteine by cystathionine-γ-lyase, cystathionine-β-synthase or 3-mercaptopyruvate sulfurtransferase. Enzymatically generated H_2_S can either be stored as sulfane sulfur (oxidative formation of protein polysulfides)^12,13^; or degraded by the sulfide oxidation pathway in mitochondria^14^.

In addition to enzymatically generated H_2_S, there are exogenous sources of this gas^15^. For example, up to millimolar amounts of H_2_S can occur in the digestive tract due to food content and the activity of sulphur-metabolising microbiota^16^. Epithelia are predominantly exposed to exogenous H_2_S of environmental or microbiological origin and are challenged to maintain low, physiological H_2_S concentrations in order to prevent potential toxicity due to high levels of exogenous H_2_S^15,17^. In addition to a high H_2_S metabolising capacity in epithelial cells^17^, we recently hypothesised that epithelia use their electrolyte and liquid secreting machinery as a defence strategy in order to flush potentially harmful sources for H_2_S from the epithelial surfaces^15^.

In epithelia, H_2_S exerts pro-secretory effects by either enhancing the secretion, or decreasing the absorption of electrolytes and liquid across the epithelium^15^. In rat, guinea pig and human colon preparations, H_2_S stimulates the secretion of chloride^15^. This is either due to direct actions on ion channels and transporters within the epithelial cells^18,19^, or indirectly by stimulating enteric secretomotor neurons^20^. In rat colon epithelial cells, H_2_S induces an increase in the intracellular calcium concentration, which triggers the opening of apical calcium-sensitive chloride channels as well as calcium- and ATP-sensitive potassium channels in the basolateral epithelial cell membrane. This hyperpolarises the apical membrane potential and facilitates apical chloride efflux^18,19^. Similar observations were recently reported in a rat vaginal epithelial preparation, where exogenous H_2_S elicits a chloride secretion which involved activation of basolateral ATP-sensitive potassium channels and apical chloride efflux via CFTR^21^. However, the mechanism of how H_2_S might facilitate opening of CFTR remains elusive.

In this study we investigated the regulation of CFTR by H_2_S. Using heterologous expression of human CFTR in *Xenopus* oocytes, we provide evidence that low-micromolar H_2_S concentrations decrease phosphodiesterase activity. This activates the cAMP/PKA signalling axis and triggers activation of CFTR.

## Results

### Hydrogen sulfide stimulates CFTR in *Xenopus* oocytes

**Figure 1:**
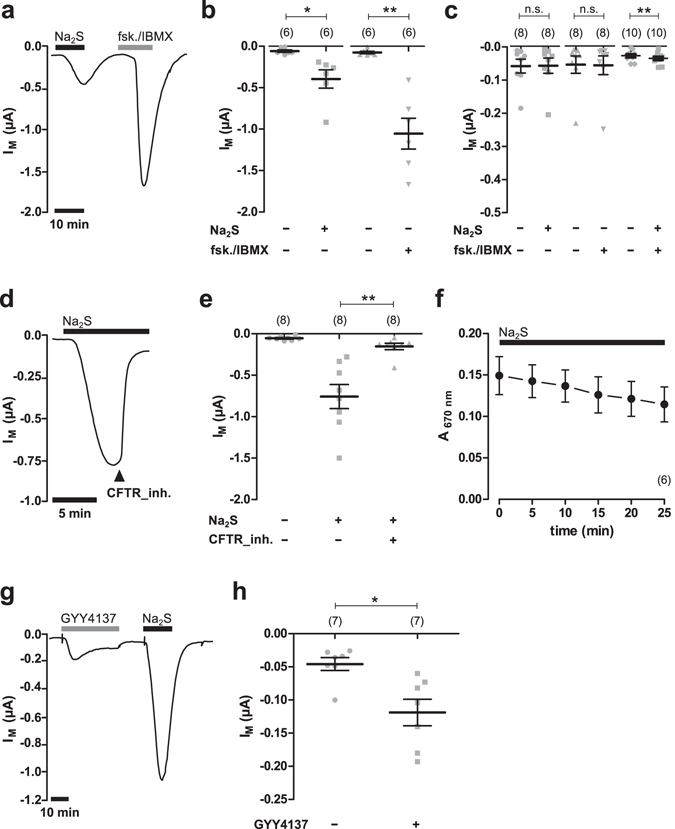
Hydrogen sulfide stimulates CFTR in *Xenopus* oocytes. (**a**) A representative current trace of a TEVC recording of a CFTR-expressing oocyte. The application of Na_2_S (50 µM, black bar) as well as forskolin (fsk.; 5 µM) and IBMX (100 µM; grey bars) led to an increase in transmembrane current signals (I_M_). (**b)** Statistical analysis of data obtained from experiments as shown in panel a. Depicted are values of I_M_ (before drug application or peak values after drug application) from individual experiments (grey symbols) as well as means ± SEM (*P ≤ 0.05, Wilcoxon signed rank test; **P ≤ 0.01, Student’s paired t-test). (**c**) Summarised data from experiments as similar to those shown in panels a and b, using native, non-CFTR-expressing oocytes. Values of I_M_ were taken at time point were CFTR-expressing oocytes of the same donor had the maximal response to the drugs. Depicted are means ± SEM (**P ≤ 0.01, Student’s paired t-test). (**d)** A representative current trace of a TEVC recording of a CFTR-expressing oocyte. After application of Na_2_S (50 µM, black bar), the CFTR inhibitor CFTR_inh172 (CFTR_inh.; 25 µM) was additionally applied. This readily inhibited values of I_M_. (**e**) Statistical analysis of data obtained from experiments as shown in panel d. Depicted are values of I_M_ (before drug application or peak values after drug application) from individual experiments (grey symbols) as well as means ± SEM (**P ≤ 0.01, Wilcoxon signed rank test). (**f**) Evaporative loss of H_2_S was measured by monitoring the concentration of H_2_S in the employed buffers solutions by the formation of methylene blue. Depicted are values for methylene blue absorbance at 670 nm over time. Na_2_S (50 µM) exposure is indicated by the black bar. (**g)** A representative current trace of a TEVC recording of a CFTR-expressing oocyte. Both, GYY4137 (500 µM, grey bar) as well as Na_2_S (50 µM, black bar) stimulated I_M_. (**h)** Statistical analysis of data obtained from experiments as shown in panel g. Depicted are values of I_M_ (peak values after drug application) from individual experiments (grey symbols) as well as means ± SEM (*P ≤ 0.05, Wilcoxon signed rank test). Numbers of experiments (n) are indicated in parentheses.

Human CFTR was heterologously expressed in *Xenopus laevis* oocytes and channel activity was measured electrophysiologically by two-electrode voltage-clamp (TEVC) microelectrode recordings. In order to investigate the potential influence of H_2_S on heterologously expressed CFTR, the H_2_S-forming sulphur salt Na_2_S was employed (Fig. 1). The application of 50 µM Na_2_S to CFTR-expressing oocytes significantly increased transmembrane currents from −0.059 ± 0.015 µA to −0.395 ± 0.11 µA (n = 6; N = 3; P = 0.0313; Fig. 1a,b). This effect was transient, as transmembrane currents began to decline in the presence of Na_2_S. Furthermore, the effect was fully reversible upon removal of Na_2_S. In order to confirm expression of CFTR, the cAMP-elevating compounds forskolin (5 µM) and IBMX (100 µM) were applied to the oocyte’s superfusate. The application of forskolin/IBMX elicited a significant increase in transmembrane current from −0.076 ± 0.015 µA to −1.055 ± 0.186 µA (n = 6; N = 3; P = 0.0030; Fig. 1a,b). Native oocytes which did not express CFTR did not respond to forskolin/IBMX (Fig. 1c). Transmembrane currents of these oocytes were −0.054 ± 0.026 µA before, and −0.056 ± 0.028 µA after application of the drugs (n = 8; N = 3; P = 0.7525). There was also no effect of Na_2_S (50 µM) on native oocytes. Transmembrane currents were −0.058 ± 0.021 µA without and −0.057 ± 0.023 µA with Na_2_S (n = 8; N = 3; P = 0.4375; Fig. 1c). However, there was a small but significant increase in transmembrane current from −0.027 ± 0.005 µA to −0.035 ± 0.006 µA when a combination of Na_2_S and forskolin/IBMX was aspplied (n = 10; N = 2; P = 0.008; Fig. 1c).

The Na_2_S-induced current of CFTR-expressing oocytes was rapidly inhibited by the additional application of the CFTR inhibitor CFTR_inh-172 (Fig. 1d,e). Under perfusion with 50 µM Na_2_S, transmembrane currents increased to −0.757 ± 0.145 µA and were rapidly inhibited to −0.153 ± 0.039 µA after addition of 25 µM CFTR_inh-172 (n = 8; N = 2; P = 0.0078). The transient nature of the current which was stimulated by Na_2_S was not the result of a rapid evaporative loss of H_2_S from the buffer solutions. Na_2_S was measured in the employed buffer solution by the formation of methylene blue and detection of its absorbance at 670 nm (Fig. 1f). There was only a minor decrease in methylene blue concentrations over time, indicating that H_2_S was present in the buffers even after a time period where current signals began to decline.

In order to confirm that the observed activation of CFTR by Na_2_S was due to H_2_S, the H_2_S-releasing compound GYY4137 which is chemically different from a sulphur salt^22^ was employed. Since GYY4137 is a slow-releasing H_2_S donor^22^, higher concentrations (500 µM) were used (Fig. 1g,h). GYY4137 elicited a small but significant activation of CFTR. Transmembrane currents of CFTR expressing oocytes significantly increased from −0.046 ± 0.010 µA to −0.119 ± 0.020 µA (n = 7; N = 2; P = 0.0156) due to application of GYY4137 (Fig. 1g,h).

In sum, these data indicate that H_2_S activates human CFTR which is heterologously expressed in *Xenopus* oocytes.

### Hydrogen sulfide stimulates CFTR activity via cAMP-mediated signalling events

The classical intracellular signalling cascade activating CFTR involves adenylyl cyclase (AC)-mediated production of cAMP and subsequent activation of protein kinase A (PKA). PKA phosphorylates the regulatory domain of CFTR and activates the channel in the presence of ATP. In order to investigate whether or not H_2_S interferes with this signalling pathway, the effect of the cAMP-elevating drugs forskolin/IBMX was evaluated with or without H_2_S.

**Figure 2:**
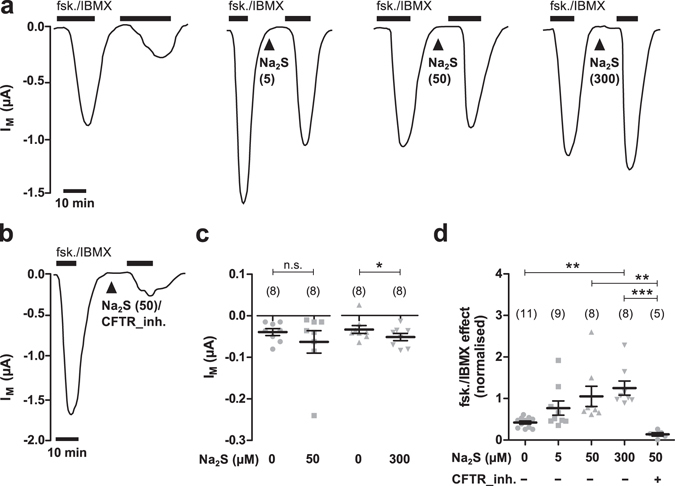
H_2_S potentiates the effect of forskolin and IBMX. (**a**) Representative current traces of TEVC recordings of CFTR-expressing oocytes. Transmembrane currents (I_M_) were recorded and oocytes were exposed twice to forskolin (fsk., 5 µM) and IBMX (100 µM, black bars) without or with application of Na_2_S (arrowheads; concentration in µM is indicated by numbers in parentheses) between the first and second stimulation with forskolin/IBMX. (**b)** Representative current trace of a TEVC recording of a CFTR-expressing oocyte. Similar to experiments shown in panel a, oocytes were stimulated twice with forskolin/IBMX and Na_2_S (50 µM) together with the CFTR inhibitor CFTR_inh172 (CFTR_inh., 25 µM) between the first and second application of forskolin/IBMX. (**c**) Statistical analysis of data obtained from experiments as shown in panel a. Depicted are values of I_M_ (peak values after drug application) from individual experiments (grey symbols) as well as means ± SEM, before and after application of Na_2_S (*P ≤ 0.05, Student’s paired t-test). (**d**) Statistical analysis of data obtained from experiments as shown in panels a and b. Depicted are normalised values from individual experiments (grey symbols) as well as means ± SEM of forskolin (fsk.)/IBMX effects. This represents the ratio of the first and second current stimulated by forskolin/IBMX (**P ≤ 0.01, ***P ≤ 0.001, Kruskall-Wallis test followed by Dunn’s Multiple Comparison Test). Numbers of experiments (n) are indicated in parentheses.

The application of forskolin/IBMX elicited a significant and transient increase in transmembrane current (Fig. 2a) from −0.064 ± 0.009 µA to −1.364 ± 0.284 µA (n = 11; N = 5; P = 0.0009). This effect was fully reversible upon removal of the drugs. A second application of forskolin/IBMX again stimulated CFTR activity from −0.081 ± 0.022 µA to −0.604 ± 0.114 A (n = 11; N = 5; P = 0.0038; Fig. 2a). The second effect of forskolin/IBMX was normalised to the effect of the first forskolin/IBMX application and defined as ‘normalised CFTR activity’. Without any additional treatment, control oocytes had thus a normalised CFTR activity of 0.42 ± 0.04 (n = 11; N = 5). We then applied increasing concentrations of Na_2_S after the first application of forskolin/IBMX (Fig. 2a). Interestingly, 50 µM Na_2_S which elicited robust currents in previous experiments (Fig. 1) did not significantly stimulate transmembrane currents after the oocytes had been exposed to forskolin/IBMX (Fig. 2a,c). Only a high dose of 300 µM Na_2_S triggered a small increase in transmembrane currents from −0.033 ± 0.010 µA to −0.051 ± 0.009 µA (n = 8; N = 3; P = 0.0423; Fig. 2a,c). However, despite the lack of an effect of Na_2_S after previous exposure of the oocytes to forskolin/IBMX, Na_2_S enhanced the second effect of forskolin/IBMX. Normalised CFTR activity dose-dependently increased due to application of Na_2_S (Fig. 2a,d). This effect was inhibited by 25 µM of CFTR_inh.172 (Fig. 2b,d). Furthermore, there was only a minor current activation due to 50 µM Na_2_S and forskolin/IBMX in native, non-CFTR expressing oocytes (Fig. 1c). These data suggest that Na_2_S potentiates CFTR-activity which was elicited by forskolin/IBMX.

**Figure 3:**
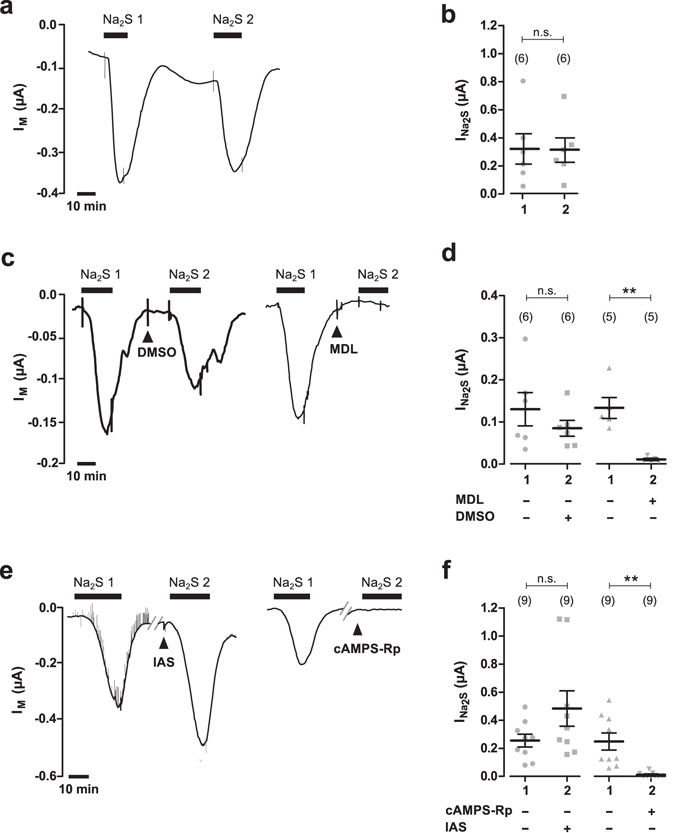
H_2_S stimulates CFTR via cAMP- and PKA-mediated signalling. (**a**) Representative current trace of a TEVC recordings of a CFTR-expressing oocyte. Transmembrane currents (I_M_) were recorded and the oocyte was exposed twice to Na_2_S (50 µM, black bar). (**b**) Statistical analysis of data obtained from experiments as shown in panel a. Depicted are values of the first (1) and second (2) Na_2_S-induced current (I_Na2S_) from individual experiments (grey symbols) as well as means ± SEM (n.s. = not significant, Student’s paired t-test). I_Na2S_ was calculated by subtracting the current before application of Na_2_S from the peak value after application of Na_2_S, resulting in positive values for I_NA2S_. (**c**) Representative current traces of TEVC recordings of CFTR-expressing oocytes. Transmembrane currents (I_M_) were recorded and oocytes were exposed twice to Na_2_S (50 µM, black bar) DMSO (0.1%; left trace) or the AC inhibitor MDL 12330 A (MDL, 20 µM; right trace) were applied between the first and second stimulation with Na_2_S (black arrowheads). (**d)** Statistical analysis of data obtained from experiments as shown in panel a. Depicted are values of the first (1) and second (2) Na_2_S-induced current (I_Na2S_) from individual experiments (grey symbols) as well as means ± SEM (**P ≤ 0.01, Student’s paired t-test). I_Na2S_ was calculated by subtracting the current before application of Na_2_S from the peak value after application of Na_2_S, resulting in positive values for I_NA2S_. (**e)** Representative current traces of TEVC recordings of CFTR-expressing oocytes. Transmembrane currents (I_M_) were recorded and oocytes were exposed twice to Na_2_S (50 µM, black bar). The perfusion recording was stopped briefly between the first and second stimulation with Na_2_S (grey lines). Then, an intracellular-analogous solution (IAS) or IAS containing the PK-antagonist cAMPS-Rp (87 µM) were injected into the oocytes before the second stimulation with Na_2_S (black arrowheads). (**f)** Statistical analysis of data obtained from experiments as shown in panel a. Depicted are values of the first (1) and second (2) Na_2_S-induced current (I_Na2S_) from individual experiments (grey symbols) as well as means ± SEM (**P ≤ 0.01, Wilcoxon signed rank test). Numbers of experiments (n) are indicated in parentheses.

To investigate if the Na_2_S-induced stimulation of CFTR involves AC and PKA, specific inhibitors of these enzymes were employed and Na_2_S-induced currents (I_Na2S_) were estimated with or without these drugs (Fig. 3). Na_2_S (50 µM) was applied twice to CFTR-expressing oocyte in order to control for a potential desensitisation in response to repetitive Na_2_S-exposure (Fig. 3a,b). The first I_Na2S_ was 0.321 ± 0.108 µA and not significantly different from the second I_Na2S_ which was 0.312 ± 0.087 µA (Fig. 3b; n = 6; N = 5; P = 0.854).

MDL 12330 A was used as an inhibitor of AC. In control experiments, Na_2_S (50 µM) was applied to CFTR-expressing oocytes, which led to an I_Na2S_ of 0.130 ± 0.040 µA (n = 6; N = 2). Afterwards, the oocytes were perfused with DMSO (0.1%; the solvent for MDL 12330A) and stimulated again with 50 µM Na_2_S. This resulted in an I_Na2S_ of 0.085 ± 0.019 µA (n = 6; N = 2), which was not significantly different (P = 0.1395) from the first I_Na2S_ (Fig. 3c,d). By contrast, when MDL 12330A was applied, I_Na2S_ significantly decreased from 0.133 ± 0.035 µA to 0.011 ± 0.003 µA (n = 8; N = 2; P = 0.0056; Fig. 3c,d).

A similar experiment was performed with cAMPS-Rp – a PKA antagonist – which was directly injected into the oocytes during experiments. For control experiments, CFTR-expressing oocytes were stimulated with Na_2_S (50 µM). Subsequently, 9.2 nl of an intracellular-analogous solution (IAS) was injected into the oocytes and the cells were stimulated a second time with Na_2_S. This manoeuvre increased (although values did not reach statistical significance) I_Na2S_ from 0.255 ± 0.046 µA to 0.484 ± 0.126 µA (n = 9; N = 2; P = 0.0743; Fig. 3e,f). A similar observation has been reported in a previous study^23^, where a volume-increase in oocytes increased the activation of CFTR by cAMP-elevating compounds. By contrast, injection of the PKA antagonist cAMPS-Rp abrogated the second effect of Na_2_S. Values of I_Na2S_ significantly decreased from 0.248 ± 0.061 µA to 0.011 ± 0.006 µA (n = 9; N = 2; P = 0.0039; Fig. 3e,f). Taken together, these data show that H_2_S activates CFTR by cAMP- and PKA-mediated signalling in *Xenopus* oocytes.

### Hydrogen sulfide targets phosphodiesterase rather than adenylyl cyclase

An increase in intracellular cAMP concentrations could either be the result of enhanced cAMP production by AC, or inhibition of cAMP degradation by phosphodiesterase (PDE). H_2_S might thus stimulate AC or inhibit PDE – both effects would result in accumulation of cAMP, a downstream activation of PKA and subsequent stimulation of CFTR. In order to discriminate between AC- and PDE-mediated contributions to CFTR activation, CFTR was stimulated with maximal effective concentrations of either forskolin (AC activator) or IBMX (PDE inhibitor). If H_2_S potentiated the effect of forskolin, but not that of IBMX, H_2_S likely prevents cAMP degradation by PDE. If H_2_S potentiated the effect of IBMX, but not that of forskolin, H_2_S likely stimulates cAMP production by AC.

**Figure 4:**
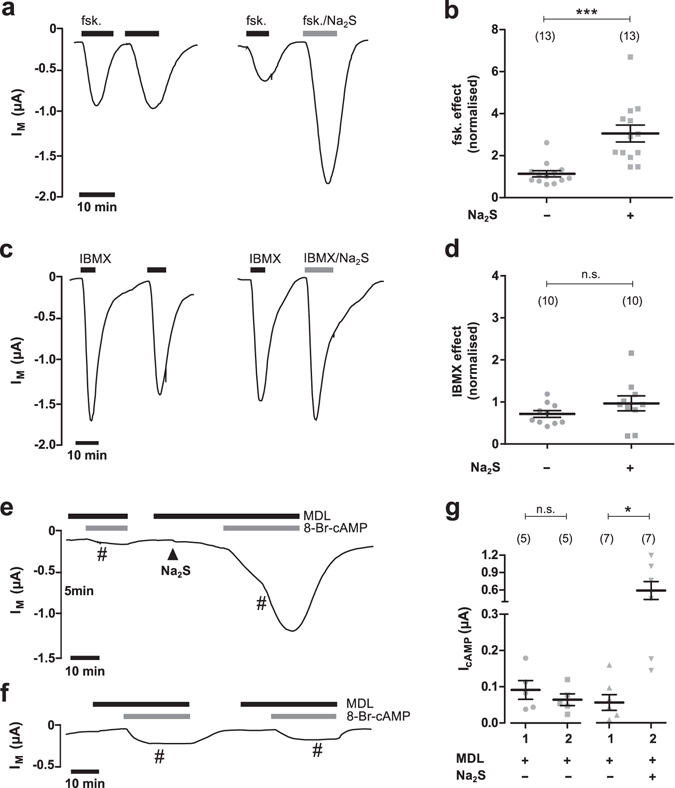
H_2_S targets phosphodiesterase rather than adenylyl cyclase. (**a**) Representative current traces of TEVC recordings of CFTR-expressing oocytes. Transmembrane currents (I_M_) were recorded and oocytes were either exposed twice to forskolin (fsk., 5 µM; black bars; left trace) or first to forskolin and then to a combination of forskolin and 50 µM Na_2_S (grey bar; right trace). (**b**) Statistical analysis of data obtained from experiments as shown in panel a. Depicted are normalised values from individual experiments (grey symbols) as well as means ± SEM of forskolin (fsk.) effects. This represents the ratio of the first and second current stimulated by forskolin (***P ≤ 0.001, Mann-Whitney test). (**c**) Representative current traces of TEVC recordings of CFTR-expressing oocytes. Oocytes were either exposed twice to IBMX (1 mM; black bars; left trace) or first to IBMX and then to a combination of IBMX and 50 µM Na_2_S (grey bar; right trace). (**d**) Statistical analysis of data obtained from experiments as shown in panel c. Depicted are normalised values from individual experiments (grey symbols) as well as means ± SEM of IBMX effects. This represents the ratio of the first and second current stimulated by IBMX Student’s unpaired t-test with Welch’s correction). (**e, f**) Representative current traces of TEVC recordings of CFTR-expressing oocytes. (**f**) Oocytes were exposed twice to membrane-permeable 8Br-cAMP (100 µM, grey bars) in the presence of the AC-inhibitor MDL 12330 A (MDL, 20 µM; black bars). The perfusion was stopped (indicated by the number symbol’) when 8-Br-cAMP was in the perfusion chambers in order to avoid massive consumption of this compound. Perfusion was started again at the time of drug removal. (**e**) The same protocol was employed, with the exception that Na_2_S (50 µM, black arrowhead) was applied before the second exposure to 8Br-cAMP. (**g**) Statistical analysis of data obtained from experiments as shown in panels e and f. Depicted are values of the first (1) and second (2) 8-Br-cAMP-induced current (I_cAMP_) from individual experiments (grey symbols) as well as means ± SEM (**P ≤ 0.01, Student’s paired t-test). Numbers of experiments (n) are indicated in parentheses.

First, we investigated if H_2_S affects the effect of forskolin alone (Fig. 4a). Since we were not able to additionally stimulate CFTR activity by increasing the forskolin concentration to 30 µM (data not shown), we considered the employed concentration of 5 µM as maximally effective, an observation which is consistent with a reported EC_50_ value of ~0.07 µM for forskolin in airway epithelia^24^. In control experiments, forskolin (5 µM) was applied to CFTR-expressing oocytes, which stimulated I_M_ by 0.324 ± 0.056 µA (n = 13; N = 3). After wash-out, the oocytes were stimulated again with 5 µM forskolin. This resulted in a second stimulation of I_M_ by 0.363 ± 0.068 µA (n = 13; N = 3; Fig. 4a). The second effect of forskolin was normalised to the effect of the first forskolin application and defined as ‘normalised forskolin effect’ (Fig. 4b). Under these control conditions, the normalised forskolin effect was 1.135 ± 0.146 (n = 13; N = 2). By contrast, oocytes which were treated with 50 µM Na_2_S (together with the second application of forskolin) had a significantly enhanced normalised forskolin effect of 3.054 ± 0.405 (n = 13, N = 3, P < 0.0001, Gaussian approximation; Fig. 4a,b).

An identical protocol was employed with a high concentration of the PDE inhibitor IBMX (1 mM) and a ‘normalised IBMX effect’ was estimated (Fig. 4c,d). The normalised IBMX effect was 0.714 ± 0.080 (n = 10; N = 3) under control conditions, and not significantly different from that which was estimated in the presence of Na_2_S which was 0.966 ± 0.178 (n = 10; N = 3; P = 0.2208). Na_2_S thus enhanced the effect of the AC-agonist forskolin, but not that of the PDE-inhibitor IBMX. H_2_S might therefore impair PDE-mediated cAMP degradation rather than AC-mediated cAMP production.

In order to confirm these observations a different strategy was employed (Fig. 4e–g). AC-mediated cAMP-production was blocked by application of the AC inhibitor MDL 12330 A (20 µM) to CFTR-expressing oocytes. Subsequently, 100 µM of membrane-permeable 8-Br-cAMP was applied. This stimulated an increase in transmembrane current (I_cAMP_) of 0.056 ± 0.0221 µA (n = 7; N = 4). After washout of all drugs, MDL 12330 A (20 µM) was applied again and 50 µM Na_2_S was added. Afterwards, 8Br-cAMP was additionally applied and I_cAMP_ significantly increased to 0.590 ± 0.154 µA (n = 7; N = 4; P = 0.0124; Fig. 4e,g). By contrast, there was no difference between the first and second I_cAMP_ (0.091 ± 0.026 µA and 0.064 ± 0.016 µA; n = 5; N = 2; P = 0.1561) when the procedure was repeated without Na_2_S (Fig. 4f,g).These data indicate that H_2_S enhances the efficacy of 8-Br-cAMP.

In sum, these data provide evidence that H_2_S inhibits endogenous PDE in *Xenopus* oocytes. This results in cAMP-mediated stimulation of CFTR-activity via downstream activation by PKA.

## Discussion

In this study we investigated the regulation of CFTR by H_2_S. Previous studies using the mouse hippocampal cell line HT22^25^ or rat vaginal epithelial preparations^21^ suggested that CFTR might be a target for H_2_S. In order to elaborate on this hypothesis, human CFTR was heterologously expressed in *Xenopus* oocytes and functional CFTR expression was confirmed by application of the cAMP-elevating compounds forskolin and IBMX, which resulted in a transient increase in transmembrane currents which did not occur in native, non-injected oocytes. These observations are consistent with previously published functional electrophysiological analyses of human CFTR in *Xenopus* oocytes^23,26^. The H_2_S-liberating sulphur salt Na_2_S elicited a transient current stimulation of CFTR-expressing oocytes which was readily inhibited by the CFTR inhibitor CFTR_inh172 and did not occur in native oocytes. Furthermore, the slow-releasing H_2_S-liberating molecule GYY4137 also stimulated transmembrane currents in *Xenopus* oocytes. These data indicate that H_2_S, released from Na_2_S or GYY4137, stimulates CFTR activity.

We then elaborated on the signalling mechanisms which mediate the H_2_S-induced activation of CFTR. We first stimulated CFTR-expressing oocytes with forskolin/IBMX and after removal of these drugs, cells were exposed to H_2_S. Interestingly, a direct response to H_2_S only occurred when high concentrations of Na_2_S were employed. When forskolin/IBMX were applied twice to CFTR-expressing oocytes, the second activation by forskolin/IBMX was ~60% smaller than the first one. This is consistent with previous reports^23^ and can be explained by a desensitisation in response to compounds which lead to a strong increase in intracellular cAMP^26^. The lack of a pronounced effect of H_2_S after initial stimulation with forskolin/IBMX is likely explained by the fact that Na_2_S alone is a much weaker stimulator of CFTR activity than forskolin/IBMX (as shown in Fig. 1a) and its effect is lost within the desensitisation after exposure to these compounds. Nevertheless, we found that H_2_S potentiated the second effect of forskolin and IBMX, indicating that the effect of H_2_S is additive to that of forskolin/IBMX. Furthermore, the H_2_S-induced activation of CFTR was lost in the presence of the AC inhibitor MDL12330A as well as the PKA antagonist cAMPS-Rp. These data indicate that H_2_S activates CFTR via the cAMP/PKA signalling axis, a finding which is consistent with a previous study demonstrating an increase in intracellular cAMP concentrations by H_2_S in *Xenopus* oocytes^27^. H_2_S is membrane-permeable^28^ and might target AC in order to stimulate cAMP production. It has been previously shown that H_2_S either stimulates^27,29^ or inhibits^30,31^ AC activity, indicating that this enzyme represents indeed a molecular target for H_2_S. However, we found that H_2_S was able to potentiate the stimulation of CFTR by the AC-activator forskolin, but not by a high concentration (1 mM) of the PDE inhibitor IBMX. If H_2_S was acting on AC, there should be an additional effect over that of PDE inhibition alone – irrespective of the concentration of the PDE inhibitor. These data therefore suggest that H_2_S targets degradation rather than production of cAMP. However, these experiments alone do not justify a definite conclusion on the target for H_2_S. Based on the experiment using forskolin alone, a potential effect of H_2_S on AC cannot be ruled out completely. Forskolin increases the affinitiy of two cytoplasmic domains (C1 and C2) of AC for each other and enhances its activity^32^. In the presence of forskolin, AC becomes more sensitive to e.g. G_sα_^32^, suggesting that it is possible that there is a synergistic activation of AC by H_2_S in the presence of forskolin, but not in the presence of IBMX.

In addition to inhibiting PDEs, IBMX inhibits adenosine receptors, including endogenous adenosine receptors in *Xenopus* oocytes. Activation of these receptors inhibits AC and antagonising the receptor with IBMX might therefore elevate cAMP-concentrations – irrespective of the block of PDE-mediated degradation of cAMP. However, as shown in a study by Kobayashi *et al*. the receptors need to be activated by a ligand (adenosine) in order to generate a current signal in oocytes and this does not occur with a perfusion system as employed in our study^33^. Furthermore, it is not possible to discriminate between cGMP- and cAMP-PDEs using IBMX. It is possible that cGMP activates CFTR in *Xenopus* oocytes^34^, however, if H_2_S acted via cGMP, the effects of Na_2_S should not be sensitive to the adenylyl cyclase inhibitor MDL. This is further confirmed by the fact that H_2_S potentiated the stimulation of CFTR by exogenous membrane-permeable 8-Br-cAMP under conditions where endogenous cAMP production was blocked by the AC inhibitor MDL 12330A. The increased CFTR stimulation by 8-Br-cAMP in the presence of H_2_S can only be explained by impaired degradation of 8-Br-cAMP by endogenous PDE activity. Consistent with this hypothesis, several studies demonstrated that H_2_S is able to inhibit cAMP and cGMP degradation by PDEs^35–37^.

Most interestingly, small amounts (nano- to lower micro-molar range) of the sulphur salt NaHS inhibited PDE-mediated cAMP breakdown in cell-free systems^35,37^, suggesting that H_2_S might directly interfere with PDE activity – independently of cellular signalling cascades. This might be the result of an interference of H_2_S with zinc, thereby reducing the activity of zinc-dependent PDE^35^. Alternatively, H_2_S might target cysteine residues in the enzyme by persulfidation^35^. However, H_2_S alone cannot modify thiol groups^38^, whereas derivatives of H_2_S such as polysulfides^13^ or nitroxyl, which might be formed in the cytoplasm of cells, are able to do so^38^. The precise mechanisms how H_2_S regulates PDE function remain to be elucidated; however, the *Xenopus* oocyte might be a useful model in order to address these questions.

In sum, we provide evidence that H_2_S inhibits endogenous PDE in the *Xenopus* oocyte, which likely results in an accumulation of cAMP and downstream activation of CFTR via PKA. This scenario, however, requires a constitutive production of cAMP in these cells. *Xenopus* oocytes are arrested in the G2 stage of meiosis I^39^. The G2 arrest is maintained by AC-mediated production of cAMP which is believed to prevent oocyte maturation via PKA-mediated signalling events^39–41^. Hence, there is a constitutive production of cAMP in *Xenopus* oocytes. Furthermore, there is an endogenous PDE activity in these cells^42^ and the activities of both AC as well as PDE determine the concentration of cAMP. When *Xenopus* oocytes are exposed to progesterone, cAMP levels decrease, the cells undergo nuclear membrane breakdown (GVBD) and mature into a fertilisable egg^39^. This process is inhibited by IBMX^42^, again demonstrating that inhibition of PDE activity leads to an increase in the concentration of cAMP. Consistently, IBMX as well as H_2_S alone were able to stimulate CFTR activity in these cells in the present study.

Our data are consistent with an emerging body of evidence that H_2_S targets the cAMP-signalling axis. Whereas this study and others^27,29^ provide evidence for an activation of the cAMP-pathway by H_2_S, inhibition of cAMP-signalling has also been reported^43–45^. A study by Lu *et al*. demonstrated inhibition of AC and stimulation of PDE by NaHS in AS4.1 cells^44^. These inconsistent findings suggest that the net-effect of H_2_S on cAMP signalling depends not only on whether AC or PDE is targeted by H_2_S, but also on the precise molecular repertoire of the cAMP-regulating machinery. In humans, ten isoforms of AC have been identified and there are 11 members of the PDE protein family which can generate nearly 100 different subtypes^46^. The specific isoform expression in a cell might critically determine whether H_2_S activates or inhibits cAMP-signalling. This is especially important, since H_2_S will diffuse across cell membranes in an unspecific manner and hence specificity is likely not achieved by membrane receptors but possibly by the subtype repertoire of intracellular signalling molecules.

In epithelia, H_2_S exerts pro-secretory or anti-absorptive effects^15,47^ and we recently suggested a concept by which epithelia use their electrolyte and liquid transport machinery as a defence mechanism in order to flush potential sources for harmful amounts of H_2_S from the epithelial surfaces^15^. The herein reported cAMP-mediated stimulation of CFTR activity would be consistent with a pro-secretory action on chloride-secreting epithelia. A recent study demonstrated a CFTR-mediated chloride secretion across rat vaginal epithelial preparations^21^. The authors speculated that this might be due to an increase in cAMP^21^ and our findings would support this hypothesis. Since H_2_S is not directly targeting CFTR, the PDE repertoire of epithelial cells will determine whether or not H_2_S triggers CFTR-mediated electrolyte secretion.

Aside from the pro-secretory effects, H_2_S prevents liquid absorption by sodium-transporting epithelia^48–50^. In these epithelia, cAMP/PKA signalling stimulates sodium absorption by an increase in the membrane abundance of sodium transporting molecules such as the epithelial sodium channel (ENaC)^51,52^. Interestingly, we recently showed that H_2_S prevents this cAMP-mediated increase in ENaC abundance in sodium-absorbing lung epithelial cells by yet unidentified mechanisms^50^. Furthermore, H_2_S did not enhance cAMP-mediated chloride secretion in primarily sodium absorbing pig airway surface epithelia (data not shown). Nevertheless, this illustrates that – depending on the specific enzymatic repertoire of the cAMP signalling axis – H_2_S might trigger cAMP-mediated electrolyte secretion in a fraction of epithelial cells, whilst simultaneously preventing enhanced electrolyte absorption in other cells. The herein reported data thus provide a step further in understanding the mechanisms of how H_2_S elicits a switch from absorptive to secretory electrolyte and water transport in epithelia.

## Methods

### Isolation of *Xenopus laevis* oocytes

All animal experiments were performed in accordance with the German animal welfare law and had been declared to the Animal Welfare Officer of the University (Registration No.: M_ 478 and M_549). The animal housing facility was licensed by the local authorities (Az: FD 62 - §11 JLU Tierphysiologie). The methods used to euthanize the animals humanely were consistent with the recommendations of the AVMA Guidelines for the Euthanasia of Animals. All procedures and experimental protocols were approved by the Animal Welfare Officer of the University as well as the regional council of Giessen (Registration No.: M_ 478 and M_549).

Oocytes of stages V/VI were isolated from freshly dissected *Xenopus laevis* ovaries and defolliculated exactly as previously described^53^. Isolated oocytes were stored at 16 °C in an oocyte culture solution containing 90 mM NaCl, 1 mM KCl, 2 mM CaCl_2_, 5 mM 4-(2-hydroxyethyl)-1-piperazineethanesulfonic acid (HEPES), 2.5 mM sodium pyruvate, 0.06 mM penicillin G and 0.02 mM streptomycin sulfate at pH 7.4.

### CFTR-cRNA synthesis and injection into oocytes

The plasmid construct for human CFTR (in pGEM-HE) was a kind gift from Professor Blanche Schwappach (University of Göttingen, Germany). Plasmids were linearised with Mlu1 (Promega, Mannheim, Germany) and subsequently *in vitro* transcribed with the RiboMAX Large Scale RNA Production System (Promega) using T7 RNA polymerase. CFTR-cRNA was diluted with diethyl pyrocarbonate (DEPC)-treated water to a final concentration of 250 ng/µl. Fifty-one nanoliters of CFTR-cRNA were injected into oocytes with a Nanoliter-Injector (Drummond Scientific, Broomall, Pennsylvania, USA) yielding final concentrations of 12.5 ng RNA/oocyte. Injected oocytes were cultured for 2–5 days at 16 °C in the oocyte culture solution.

### Microelectrode recordings (Two-Electrode Voltage-Clamp, TEVC)

CFTR-expressing oocytes were placed in a Lucite chamber which was continuously perfused with oocyte ringer solution (ORS) containing 90 mM NaCl, 1 mM KCl, 2 mM CaCl_2_, 5 mM HEPES at pH 7.4. Chlorinated silver wires served as recording electrodes and were mounted into borosilicate glass capillaries (Hilgenberg, Malsfeld, Germany) with an outer diameter of 1.2 mm, which were pulled to microelectrodes with a DMZ universal puller (Zeitz-Instruments, Martinsried, Germany) and filled with 1 M KCl. Ag/AgCl wires were used as reference electrodes and placed directly into the recording chamber. The membrane voltage was clamped to −60 mV using a TEVC amplifier (Warner Instruments, Hamden, Connecticut, USA). Transmembrane currents (I_M_) were low-pass filtered at 1000 Hertz (Frequency Devices 902, Haverhill, Massachusetts, USA) and continuously recorded with a strip chart recorder (Kipp&Zonen, Delft, The Netherlands).

### Determination of evaporative loss of H_2_S from buffer solutions

The equilibration of H_2_S with its concentration in air will eventually lead to evaporative loss of this gas from the experimental buffer solutions^54^. Therefore the relative concentration of H_2_S in ORS was determined at various time points after administration of 50 µM Na_2_S by the formation of methylene blue. Samples (300 µl) of the solutions were mixed with 500 µl of 4% zinc acetate and incubated on ice for at least 30 min. Afterwards, 200 µl of 0.1% dimethylphenylendiamine sulfate (in 5 M HCl) and 100 µl of 50 mM FeCl_3_ were added. Samples were vortexed, centrifuged at 5000× g and incubated for 5 min at room temperature. The absorption of methylene blue was measured at 670 nm with a Vis-spectrophotometer (Kruess Optronic, Hamburg, Germany).

### Chemicals and solutions

In order to apply H_2_S, the sulfur salt Na_2_S (Sigma, Taufkirchen, Germany) or the slow releasing H_2_S donor GYY4137 (Santa Cruz, Biotechnology, Dallas, Texas, USA) were employed. Na_2_S was prepared as a stock solution of 50 mM in ORS freshly before experiments and immediately diluted to final working concentrations in order to prevent evaporative loss of H_2_S from the experimental solutions. Stock solutions of 100 mM GYY4137 were prepared in H_2_O and stored at −20 °C. Forskolin (MoBiTec, Göttingen, Germany) was used as a stimulator of adenylyl cyclase and stock solutions of 10 mM were prepared in dimethyl sulfoxide (DMSO, Sigma) and stored at −20 °C. The phosphodiesterase inhibitor 3-isobutyl-1-methylxanthine (IBMX; Sigma, Taufkirchen, Germany) was dissolved to 100 mM in DMSO and stored at +4 °C. The adenylyl cyclase inhibitor MDL 12330 A hydrochloride (MDL; Tocris Bioscience, Bristol, UK) was dissolved to 20 mM in DMSO and stored at +4 °C. cAMPS-Rp (Tocris Bioscience) was used as a competitive antagonist of cAMP-induced Protein Kinase A (PKA). Stock solutions of cAMPS-Rp were prepared to 10 mM in H_2_O and stored at −20 °C. cAMPS-Rp was injected into *Xenopus* oocytes during TEVC experiments. Therefore, stock solutions of cAMPS-Rp were diluted 1:1 with an intracellular-analogous solution (IAS) which contained 20 mM NaCl, 130 mM KCl, 2 mM MgCl_2_ and 5 mM HEPES at pH 7.3. This mixture (9.2 nl) was injected into oocytes, leading to concentrations of ~87 µM cAMPS-Rp per oocyte. Corresponding control experiments were performed with IAS. The membrane permeable cAMP-analogue 8-Br-cAMP (Tocris Bioscience) was prepared as a 10 mM stock solution in H_2_O and stored at −20 °C.

### Drug application

Drugs were generally applied using a gravity-driven perfusion system. In order to reduce the amount of drugs needed, the membrane-permeable cAMP-analogue 8-Br-cAMP was washed into the perfusion chamber and the perfusion was stopped afterwards. The PKA inhibitor cAMPS-Rp was directly injected into the oocytes since this strategy was established earlier and achieved adequate inhibition of PKA^55^.

### Statistical analysis

For electrophysiological transmembrane recordings, outward-anion currents are defined as negative current signals and depicted in all figures as downward-deflections of the current traces. Data are presented as individual data points (grey symbols) as well as means ± standard error of the mean (SEM). The number of individual oocytes is indicated with ‘n’, whereas the number of donor frogs is represented by ‘N’. Statistical analysis of data was performed with GraphPad Prism version 5 (La Jolla, California, USA). Data were analysed for normal distribution by the Kolmogorov-Smirnov test. For paired experiments, Student’s paired *t*-test or non-parametric Wilcoxon matched-pairs test (two-tailed) were used. Independent experiments were compared with Student’s unpaired t-test or non-parametric Mann-Whitney test (two-tailed). Mutiple comparison analysis was performed by Kruskall-Wallis test followed by Dunn’s Multiple Comparison Test. P-values ≤ 0.05 were regarded as statistically significant and marked with an asterisk (*). P-values ≤ 0.01 and ≤0.001 were marked with “**” and “***”, respectively.
