# Publisher Correction: Hydrogen sulfide stimulates CFTR in *Xenopus* oocytes by activation of the cAMP/PKA signalling axis

**DOI:** 10.1038/s41598-018-21979-6

**Published:** 2018-03-08

**Authors:** Alexander Perniss, Kathrin Preiss, Marcel Nier, Mike Althaus

**Affiliations:** 1 Institute for Animal Physiology, Justus-Liebig-University, Giessen, Germany; 2 Present Address: Institute for Anatomy and Cell Biology, Justus-Liebig-University, Giessen, Germany; 3 School of Biology, Newcastle University, Newcastle upon Tyne, United Kingdom

Correction to: *Scientific Reports* 10.1038/s41598-017-03742-5, published online 14 June 2017

The original PDF version of this Article contained an error where Figure 1 was truncated during publication. In addition, this Article contained an error in the legend of Figure 1 in the original HTML and PDF versions, where:

“(h) Statistical analysis of data obtained from experiments as shown in panel d.”

now reads:

“(h) Statistical analysis of data obtained from experiments as shown in panel g.”

These errors have now been corrected in the HTML and PDF versions of this Article.

